# Evaluation of the safety and efficacy of low-dose rasburicase in critically ill children with haematological malignancies

**DOI:** 10.1007/s11096-020-01144-8

**Published:** 2020-09-24

**Authors:** Yuxin Pei, Yu Li, Yujian Liang, Lingling Xu, Xueqiong Huang, Yijuan Li, Wen Tang, Xiaoyun Jiang

**Affiliations:** The First Affiliated Hospital of Sun-yat, Sen University, Guangzhou, Guangdong Province China

**Keywords:** Children, Haematological malignancies, Hyperuricaemia, Intensive care unit, Rasburicase

## Abstract

*Background* The recommended dose of rasburicase is quite expensive, thus limiting its use. Whether a lower dose of rasburicase would be equally effective for critically ill children, who often have more complicated situations and a higher risk of hospital death, is still unknown. *Objective* To explore the safety and efficacy of low-dose rasburicase in critically ill children with haematological malignancies who are at high risk of tumour lysis syndrome. *Setting* A single-centre retrospective cohort study. *Method* Children with haematological malignancies who had a history of rasburicase exposure during an intensive care unit stay were enrolled. Patients were divided into two groups according to the initial dosage of rasburicase: the standard-dose group (> 0.1 mg/kg/day) and the low-dose group (≤ 0.1 mg/kg/day). The adverse events and short-term prognosis of the two groups were compared. *Results* Thirty-seven children were selected, 22 in the standard-dose group and 15 in the low-dose group. The most common tumour type was Burkitt’s lymphoma (81%), followed by acute lymphoblastic leukaemia (11%). All patients were at high risk of tumour lysis syndrome, and 73% of them had 3 or more tumour lysis syndrome risk factors. The uric acid levels of 90% of patients with hyperuricaemia returned to the normal range within 12 h (100% in the standard-dose group and 75% in the low-dose group, *P* = 0.083). Eighty-four percent of patients presented serious complications, including tumour lysis syndrome (73%), acute kidney injury (59%), renal replacement treatment (24%), respiratory failure (24%), disseminated intravascular coagulation (16%) and heart failure (11%). There was no significant difference in the incidence of serious complications between the two groups. The overall 7-day and 28-day survival rates after intensive care unit admission were 86% and 84%, respectively. The average length of stay in the intensive care unit was 9.92 ± 5.13 days. Neither the short-term mortality nor the length of stay in the intensive care unit were significantly different between the two groups. *Conclusion* Low-dose rasburicase is effective and may be an acceptable choice for critically ill children with haematological malignancies.

## Impacts on Practice

- Rasburicase is recommended for children who are at high risk of tumour lysis syndrome.
- This research provides guidance on using rasburicase in children with a high tumour burden managed in the intensive care unit.
- Dosage regimes of < 0.1 mg per kg body weight per day resulted in similar outcomes compared to dosage regimens of > 0.1 mg/kg body weight per day
- Finally, this study may provide novel study angles for dosage regimens of rasburicase, especially in resource-limited regions.

## Introduction

Tumour lysis syndrome (TLS) is an important cause of early death as well as admission to the intensive care unit (ICU) in children with newly diagnosed haematologic malignancies [1]. It is a serious metabolic syndrome that occurs due to rapid lysis of tumour cells. Lysed cells release large quantities of cellular contents into the blood circulation and may result in hyperkalaemia, hypocalcaemia, hyperuricaemia, hyperphosphatemia and multiple organ dysfunction [2]. Both hyperuricaemia and hyperphosphatemia can cause the deposition of crystals in the renal tubular system. This deposition may result in acute kidney injury (AKI), a serious complication associated with a poor short-term prognosis in children [3]. Children with haematologic malignancies have a high potential for cell lysis; thus, they are considered to have a high risk of developing TLS, and combined treatment regimens, including close laboratory and clinical monitoring, increased hydration and uric acid control therapy, have been proposed.

In the treatment of hyperuricaemia, urate oxidase is recommended for children at high risk of TLS because it can rapidly convert uric acid to allantoin, which is far more soluble than uric acid. Compared with those who use allopurinol, children who use rasburicase, a type of recombinant urate oxidase, are less likely to receive renal replacement therapy. The Food and Drug Administration (FDA)-approved dose of rasburicase is 0.2 mg/kg/day for up to 5 days. In clinical practice, a dosage of 0.1–0.2 mg/kg/day of rasburicase is often applied [4]. Nonetheless, this uric acid-lowering strategy is much more expensive than the traditional treatment with allopurinol [5]. For decrease costs, some researchers attempted to determine the minimum effective dose and frequency for rasburicase. A recent study showed that a fixed dose of 6 mg rasburicase is effective for children and adolescents who have a weight exceeding 30 kg [6]. However, haematologic malignancies at high risk of TLS are also prevalent in young children [7, 8]. There is still limited evidence on the use of a lower dose of rasburicase, especially in critically ill children [9, 10]. It is therefore important to explore the efficacy of low-dose rasburicase therapy in critically ill children with haematologic malignancies.

### Aim of the study

The objective of this study was to explore the safety and efficacy of low-dose rasburicase in critically ill children with haematologic malignancies who were at high risk of TLS.

### Ethics approval

This retrospective chart review study involving human participants was in accordance with the ethical standards of the institutional and national research committee and with the 1964 Helsinki Declaration and its later amendments or comparable ethical standards. The Medical Ethics Committee of the First Affiliated Hospital of Sun Yat-sen University approved this study [No. 2020-092].

## Methods

### Study design and participants

This single-centre retrospective cohort study included children with newly diagnosed haematologic malignancies who were admitted to the Paediatric Intensive Care Unit (PICU) of The First Affiliated Hospital of Sun Yat-sen University between January 2014 and January 2020. Patients < 18 years old who had a confirmed diagnosis of haematological malignancies by histology or cytology and received at least one dose of rasburicase were eligible for inclusion. Patients who had completed induction chemotherapy or did not have rasburicase exposure during their ICU stay were excluded. Clinical information, including demographic data, tumour factors, treatment modalities, and outcomes, was collected. The definition of and assessment of risk for TLS was performed according to the British Committee for Standards in Haematology-recommended guidelines [11]. The clinical TLS risk classification proposed by Michael Darmon was used [12]. AKI was identified and staged based on the 2012 Kidney Disease Improving Global Outcome (KDIGO) definition [13]. Chemotherapy regimens were based on tumour characteristics according to the South China Children’s Cancer Group (SCCCG) protocol [7, 14]. The standard treatment protocols for TLS include risk assessment, increased hydration, careful monitoring, and management of hyperuricaemia [11]. All patients were managed with increased hydration for 3 l/m^2^/24 h to maintain urine output > 4 ml/kg/h for infants and 100 ml/m^2^/h for older children. Laboratory tests, including analyses for electrolytes, uric acid levels and renal function, were performed at least daily during the ICU stay. Two uric-acid-lowering agents were applied according to different risk stratifications: rasburicase was given prophylactically before or within the first week of initial chemotherapy for children at high risk of TLS after PICU admission, while allopurinol was given for the others. According to the daily dose of rasburicase, children were divided into two arms: the standard-dose group (> 0.1 mg/kg/day and ≤ 0.2 mg/kg/day) and the low-dose group (≤ 0.1 mg/kg/day). Follow-up information was obtained from general ward or outpatient clinic visits.

### Outcomes

The 7-day and 28-day mortality rates after admission to the PICU were used to assess short-term prognosis. The efficacy of treatments with different dosages of rasburicase was evaluated by comparing the need for renal replacement therapy (RRT), the duration of hyperuricaemia, the incidence of serious complications and the length of stay in the ICU.

### Statistical analysis

Demographic and tumour features are summarized descriptively. Continuous variables are reported as the mean ± SD or median (lower quartile: upper quartile) and analysed by two-tailed t test using a Student t test or Wilcoxon rank-sum test. Categorical variables are presented as actual numbers and percentages and compared by using the Chi square test, Fisher’s exact test or Pearson’s Chi square test as appropriate. Overall survival curves were plotted using the Kaplan-Meier method. *P* values less than 0.05 were considered significant, and actual values are shown in each table. Statistical analyses were carried out with SPSS version 16.0 and GraphPad Prism version 5.03.

## Results

### Clinical Characteristics

From January 2014 to January 2020, 65 critically ill children with newly diagnosed haematological malignancies were admitted to our centre. Twenty-eight patients who did not have rasburicase exposure were excluded. Thirty-seven patients were eligible to be included in this study; 28 were boys, and 9 were girls. The mean age at diagnosis was 6.4 ± 3.4 years, with a mean disease course of 35 ± 26 days. Most of them had Burkitt’s lymphoma (30/37, 81%). The remaining cases were of lymphoblastic lymphoma (2/37, 5%), acute lymphoblastic leukaemia (4/37, 11%) and acute myeloid leukaemia (1/37, 2%).

The main reasons for ICU admission were dyspnoea or acute respiratory failure (41%), abdominal compartment syndrome (27%) and monitoring (24%). Hyperuricaemia was present on admission in 81% of cases. The baseline lactate dehydrogenase (LDH) was 2179 (1137,3282) U/L, and the peak LDH often appeared on D3-D5 of pre-phase chemotherapy, with a median value of 2936 (1697,6497) U/L. Older children or those with higher weights preferred to take lower initial doses of rasburicase. In addition, there was no difference in baseline characteristics between the standard-dose group and the low-dose group. The demographic data and tumour characteristics of the two groups are summarized in Table 1.

**Table 1:** Baseline characteristics of critically ill children with haematological malignancies

	All patients (n = 37)	Standard-dose group (>0.1 mg/kg/day) (n = 22)	Low-dose group (≤0.1 mg/kg/day) (n = 15)	P value
Age	6.4 ± 3.4	5.1 ± 3.1	8.3 ± 2.8	0.002*
Sex				
Male	28	16	12	0.908
Female	9	6	3	
Weight	22.3 ± 10.9	17.8 ± 8.3	28.9 ± 11.0	0.001*
Disease course	35 ± 26	35 ± 27	35 ± 25	0.999
Rasburicase exposure				
Total dose (mg/kg)	0.58 ± 0.42	0.76 ± 0.41	0.33 ± 0.29	0.001*
Daily dose (mg/kg/day)	0.13 ± 0.06	0.16 ± 0.04	0.08 ± 0.02	<0.001*
Frequency (times)	4.58 ± 2.97	4.89 ± 2.98	4.13 ± 3.00	0.456
Tumour histology				
Burkitt’s lymphoma	30	19	11	0.405
Lymphoblastic lymphoma	2	1	1	
Acute lymphoblastic Leukaemia	4	1	3	
Acute myeloid Leukaemia	1	1	0	
Laboratory values at admission				
BUN (mmol/L)	4.68 ± 2.38	4.89 ± 2.74	4.38 ± 1.77	0.529
SCr (μmol/L)	55 ± 29	55 ± 33	55 ± 23	0.976
UA (μmol/L)	677 ± 288	625 ± 291	754 ± 274	0.184
LDH (U/L)	2179 (1137,3282)	1871 (1056,2768)	2805 (1265,4586)	0.234

*BUN* Blood urea nitrogen, *SCr* serum creatinine, *UA* uric acid, *LDH* lactate dehydrogenase; *P < 0.05

Based on the risk assessment for malignant disease type, all patients were at high risk of TLS, 14% (n = 5) had acute lymphoblastic leukaemia or acute myeloid leukaemia with an elevated white blood cell count (> 100×10^9^/l), and 86% (n = 32) had advanced-stage Burkitt’s lymphoma or lymphoblastic lymphomas. In addition, 97% of patients (n = 36) had other risk factors for TLS at admission, such as renal involvement (n = 3, 8%), renal dysfunction (n = 14, 38%), LDH ≥ 2 ×  upper limit of normal (ULN) (n = 36, 97%), hyperuricaemia (n = 27, 72.97%), hyperkalaemia (n = 1, 3%) and hyperphosphatemia (n = 1, 3%). According to the clinical TLS risk assessment score proposed by Michael et al^9^, 24% (n = 9) of patients were at very high risk of clinical TLS, while 56% (n = 5) of them developed clinical TLS (Table 3). The mean risk assessment score was 9.5 ± 5.1. No significant difference in scores was seen between the different treatment groups (9.0 ± 4.5 vs. 10.1 ± 6.0, *P* = 0.560).

### Rasburicase exposure

**Fig. 1:**
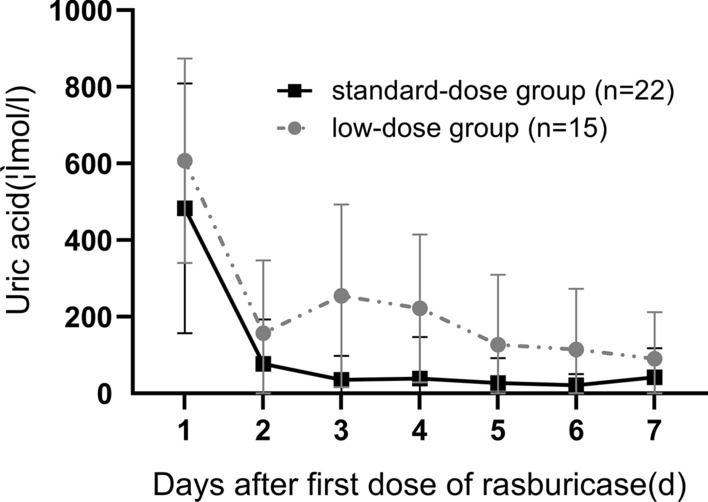
Rapid decrease in uric acid level after first dose of rasburicase in each groups. The line graph shows mean standard deviations of the uric acid decrease level

All patients received at least one dose of rasburicase, 30 cases in the pre-chemotherapy period within D-1 to D1 because of hyperuricaemia and 7 cases within D3 to D6 due to TLS or AKI during chemotherapy. Details of the rasburicase exposures are also summarized in Table 1. The mean dose of rasburicase was 0.13 ± 0.06 mg/kg/day. Twenty-two cases in the standard-dose group received dosages in the range of 0.11 mg/kg/day to 0.20 mg/kg/day, while 15 cases in the low-dose group varied from 0.04 mg/kg/day to 0.10 mg/kg/day. Eighty-one percent (30/37) of patients needed at least one more dose of rasburicase. One patient in the low-dose group who had no history of allergy or drug hypersensitivity developed anaphylactic shock after the first dose of rasburicase. No other side effects were observed during rasburicase treatment. In those patients with hyperuricaemia, the uric acid (UA) levels of 3 patients in the low-dose group decreased to normal in 36–72 h, while that of the others (27/30) returned to the normal range within 12 h. Changes in UA levels after the first dose of rasburicase are shown in Fig. 1.

### Clinical outcome

All 37 patients received pre-phase chemotherapy, as established by the South China Children’s Cancer Group (SCCCG), after histologically confirmed haematological malignancy to reduce tumour load [7, 14]. The induction treatment was adjusted in 5 patients. Three patients needed to postpone formal chemotherapy because of multiple organ dysfunction. Two patients with Burkitt’s lymphoma had delayed high-dose methotrexate treatment as a subsequent regimen due to massive ascites. The median follow-up time was 13 months (2–29 months). Clinical outcomes are listed in Table 2.

**Table 2:** Serious compilations and prognosis of critically ill children with haematological malignancies

	All patients (n = 37)	Standard-dose group (>0.1 mg/kg/day) (n = 22)	Low-dose group (≤0.1 mg/kg/day) (n = 15)	P value
Incidence of serious complications	84% (31)	77% (17)	93% (14)	0.397
TLS	73% (27)	73% (16)	73% (11)	1.000
Laboratory TLS	30% (11)	23% (5)	40% (6)	0.417
Clinical TLS	43% (16)	50% (11)	33% (5)	0.500
AKI	59% (22)	64% (14)	53% (8)	0.775
Stage 1	11% (4)	9% (2)	13% (2)	
Stage 2	16% (6)	14% (5)	7% (1)	
Stage 3	32% (12)	32% (7)	33% (5)	
Requirement of RRT	24% (9)	23% (5)	27% (4)	1.000
Respiratory failure	24% (9)	27% (6)	20% (3)	0.908
Heart failure	11% (4)	14% (3)	7% (1)	0.896
DIC	16% (6)	14% (3)	20% (3)	0.670
Others	14% (5)	9% (2)	20% (3)	0.643
Prognosis				
7-day mortality	14%	9%	20%	0.643
28-day mortality	16%	14%	20%	0.951
LOS in ICU (d)	8.95±4.77	9.41±4.71	8.27±4.93	0.482
Costs in ICU stay ($)	10003±4949	10024±3651	9971±6556	0.978

*TLS* tumour lysis syndrome, *AKI* acute kidney injury, *RRT* renal replacement therapy, *DIC* Disseminated intravascular coagulation, *LOS* length of stay, *ICU* intensive care unit

Serious complications were frequent in critically ill children with haematological malignancies (n = 31, 84%), and tumour lysis syndrome was the most common complication (n = 27, 73%). The median occurrence time of TLS was D3 (D3-D5). Hyperuricaemia (25/27) and hyperphosphatemia (17/27) were prevalent symptoms of TLS, which may lead to the high incidence of AKI. Forty-three percent of patients developed clinical TLS. The incidence of clinical TLS, stratified according to the clinical TLS risk assessment score, is shown in Table 3. There was no significant difference between the standard-dose group and the low-dose group. Fifty-nine percent of patients had AKI, 41% (9/22) of whom required renal replacement therapy (RRT), 5 in the standard-dose group and 4 in another group, with a median RRT time of 3 days. The recorded causes for haemodialysis were respiratory failure (n = 5, 2 cases combined with congestive heart failure) and severe acidosis with hyperlactatemia (n = 4). None of the surviving AKI patients required long-term haemodialysis or progressed to chronic kidney disease. The median renal function recovery time was 4.0 days (2.5–6.0 days), with no significant difference between groups (*P* = 0.802). Other serious complications, including respiratory failure (n = 9, 24%), disseminated intravascular coagulation (n = 6, 16%) and heart failure (n = 4, 11%), occurred in 27% of patients.

**Table 3:** Risk assessment and the incidence of clinical tumour lysis syndrome in critically ill children with haematological malignancies

Risk category	Score	Risk assessment of Clinical TLS			Incidence of Clinical TLS		
		All	S-D group	L-D group	All	S-D group	L-D group
Low risk	≤5	12	7	5	2	1	1
Intermediate risk	6–9	9	6	3	4	3	1
High risk	10–13	7	4	3	4	3	1
Very high risk	≥ 14	9	5	4	6	4	2

*TLS* tumour lysis syndrome, *S-D group*: standard-dose group (rasburicase > 0.1 mg/kg/day), *L-D group* low-dose group (rasburicase ≤ 0.1 mg/kg/day)

**Fig. 2:**
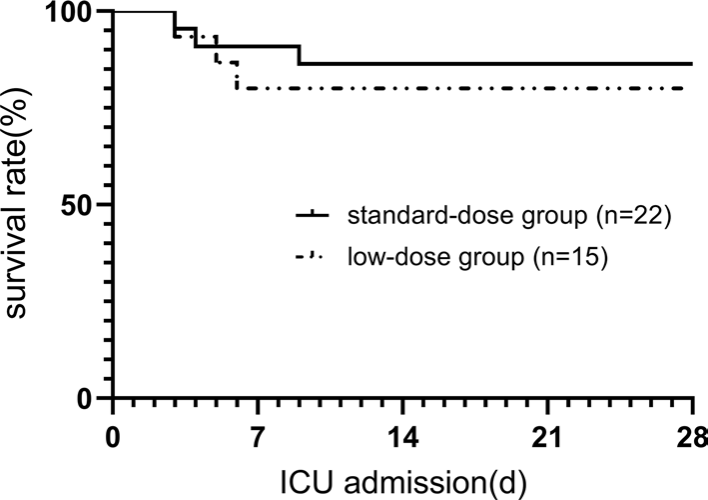
The Kaplan-Meier survival analysis for days from ICU admission to death (P = 0.610)

The overall 7-day and 28-day survival rates after ICU admission were 86% and 84%, respectively. Six patients died because of cerebral herniation (n = 3, 8%), cardiac arrest caused by clinical TLS (n = 2, 5%), and septic shock (n = 1, 3%). The other 31 patients are currently alive. The average length of stay in the ICU was 9.92 ± 5.13 days. Neither length nor cost of stay in the ICU was significantly different between the two groups. The overall survival curves of the 37 patients are shown in Fig. 2.

## Discussion

A number of studies have confirmed that rasburicase is safe and effective in the treatment of TLS in children. Although rasburicase is recommended for patients categorized as being at the highest risk of developing TLS, at present, there is still great variability in the use of rasburicase in different countries and regions. A cross-sectional study showed that the utilization rate of rasburicase exposure from 48 children’s hospitals throughout the United States was from 7% to 71% [15] and that it is estimated to be even lower in developing countries [16], far from the application recommended by the guidelines [15]. In our institution, 57% of children with haematological malignancies used rasburicase during their ICU stay. Rasburicase was used only in high-risk patients. A more stringent standard was applied in rasburicase therapy that in 97% of patients with more than one risk factor for TLS. Most patients (n = 27, 73%) even had ≥ 3 risk factors.

The recommended dose of rasburicase is 0.2 mg/kg for 5–7 days, signifying a higher cost than the cost of the standard care for TLS [5], which may be one of the reasons for its limited application. Recent studies about reducing the frequency of rasburicase administration indicated that single-dose rasburicase also has clinical efficacy and can save on drug costs in the treatment of TLS [9, 17, 18], but 21–33% of patients who are at high risk of TLS require at least two doses of rasburicase due to rebound of uric acid [10, 19]. Our study shows that the proportion of patients requiring a second dose of rasburicase appears to be higher in critically ill children. The requirement of repeated doses of rasburicase may result from the continuous disintegration of tumour cells and the production of uric acid in the early stage of chemotherapy, which lasts far longer than the duration of rasburicase action. Previous studies have shown that plasma uric acid drops to an undetectable level in 84% of patients after the first recommended dose of rasburicase [17]. Because rasburicase is expensive and a self-financed drug in our country, the expenses are beyond the financial reach of many households. Thus, we compared different dosing strategies of rasburicase to explore the optimal dosage for maximum pharmacoeconomic benefits. However, the main limitation in previous studies is that they only focused on the decline of uric acid rather than on the prognosis of the children. Information about the incidence of serious adverse events and the outcomes of such patients is indistinct, especially in critically ill children. A study on patients with malignancies who underwent chemotherapy in the ICU showed that the in-ICU incidence of TLS was 36%, much higher than that in the general ward (0.02%). AKI and the requirement of RRT are also more common in those patients, suggesting that the prevention and treatment of TLS are more challenging in ICU patients [20]. While comparing two groups with different initial doses of rasburicase, whether greater or less than 0.1 mg/kg, we found that both the incidence of severe complications and the survival rate of patients were comparable between the two groups, which suggested that a smaller initial dose of rasburicase could be considered for children in the ICU.

The incidence of TLS and AKI in this study is high, which may be related to the more complicated condition of critically ill children. In addition, the prevalence of hyperuricaemia and hyperphosphatemia may also partly explain the high incidence of AKI [21]. Nevertheless, our study shows lower mortality in critically ill patients with TLS, which may be due to the wide application of rasburicase treatment [20]. There was no difference in the incidences of TLS and AKI between different dose groups, suggesting that a low dose of rasburicase is safe and effective in critically ill children with a high tumour burden. However, further prospective studies with larger sample sizes are required to explore the efficacy of low-dose rasburicase therapy in preventing TLS and protecting renal function in critically ill children with large tumour burdens.

Prolonged ICU stays not only increase psychological stress and economic burden on the patients’ families but also make follow-up treatment difficult. A few studies have demonstrated that reducing the rasburicase dosage may reduce the cost of hyperuricaemia treatment [10, 22], but there is a lack of research concern about the total financial burden of ICU costs. In our study, no significant difference in in-ICU expenses was observed between the two groups, indicating that the reduction of the rasburicase dose not increase in-ICU expenses. This is consistent with the result in our study that low-dose rasburicase does not increase life-threatening situations. Children in the low-dose group tended to have heavier body weights; thus, the dose and cost of other drugs would increase correspondingly, which may contribute to the negative result. Although patients in the low-dose group showed a reduction trend in the in-ICU cost, more studies are needed to confirm the role of low-dose rasburicase in reducing the economic burden.

## Conclusion

Tumour lysis syndrome is a potentially life-threatening complication and one of the most common causes of short-term death in children with a large tumour burden in intensive care units. Though limited, the findings from our study indicate that using less than the recommended dose of rasburicase may be an acceptable choice for critically ill children with haematological malignancies, especially in resource-limited areas.

## Data Availability

The datasets generated during and/or analysed during the present study are available from the corresponding author on reasonable request.
